# Integrated optical probing scheme enabled by localized-interference metasurface for chip-scale atomic magnetometer

**DOI:** 10.1515/nanoph-2024-0296

**Published:** 2024-09-26

**Authors:** Jinsheng Hu, Zihua Liang, Peng Zhou, Lu Liu, Gen Hu, Mao Ye

**Affiliations:** Key Laboratory of Ultra-Weak Magnetic Field Measurement Technology, Ministry of Education, School of Instrumentation and Optoelectronic Engineering, 12633 Beihang University, Beijing 100191, China; Zhejiang Provincial Key Laboratory of Ultra-Weak Magnetic-Field Space and Applied Technology, 12633 Hangzhou Innovation Institute, Beihang University, Hangzhou 310051, China; Hangzhou Institute of Extremely-Weak Magnetic Field Major National Science and Technology Infrastructure, Hangzhou 310051, China; Hefei National Laboratory, Hefei 230088, China

**Keywords:** chip-scale quantum sensors, integrated optical probing, meta-polarizer, multi-functional metasurface, atomic magnetometer

## Abstract

Emerging miniaturized atomic sensors such as optically pumped magnetometers (OPMs) have attracted widespread interest due to their application in high-spatial-resolution biomagnetism imaging. While optical probing systems in conventional OPMs require bulk optical devices including linear polarizers and lenses for polarization conversion and wavefront shaping, which are challenging for chip-scale integration. In this study, an integrated optical probing scheme based on localized-interference metasurface for chip-scale OPM is developed. Our monolithic metasurface allows tailorable linear polarization conversion and wavefront manipulation. Two silicon-based metasurfaces namely meta-polarizer and meta-polarizer-lens are fabricated and characterized, with maximum transmission efficiency and extinction ratio (ER) of 86.29 % and 14.2 dB for the meta-polarizer as well as focusing efficiency and ER of 72.79 % and 6.4 dB for the meta-polarizer-lens, respectively. A miniaturized vapor cell with 4 × 4 × 4 mm^3^ dimension containing ^87^Rb and N_2_ is combined with the meta-polarizer to construct a compact zero-field resonance OPM for proof of concept. The sensitivity of this sensor reaches approximately 9 fT/Hz^1/2^ with a dynamic range near zero magnetic field of about ±2.3 nT. This study provides a promising solution for chip-scale optical probing, which holds potential for the development of chip-integrated OPMs as well as other advanced atomic devices where the integration of optical probing system is expected.

## 1 Introduction

Atomic sensors gain their utility through the sensitivity of atoms to external fields or perturbations and have diverse applications in a broad range of fields such as navigation [1], [2], [3], biomagnetism imaging [4], [5], [6], nuclear energy [7], gravity cartography [8] and ultralight dark matter detection [9]. Optical systems build the backbone of atomic sensors because precise control or detection of coherent free-space light is necessary to interrogate the altered quantum state of atoms in the presence of external fields. For example, optically pumped magnetometers (OPMs) based on hot alkali vapor require light with specific linear polarization and collimated wavefront to implement optical probing of polarized atomic spins [10], [11]. Optical probing allows us to detect the states of polarized atomic spins with high sensitivity based on strong light-atom interaction. Conventionally, light for optical probing is readily achieved through a combination of a convex lens and a linear polarizer (LP). In general, lens is composed of curved isotropic glass with gradual phase delays accumulated along the optical path while linear polarizer is based on the birefringence of crystals or polymers to create a phase retardation between two orthogonal polarizations, which results in conventional optical components that are bulky and challenging to integrate. In addition, optical alignment between discrete optics relies on manual assembly. Both are no longer satisfying the growing requirements for the chip-integration, mass production, and cost reduction of atomic sensors. Therefore, an efficient and multi-functional integrated optical platform is urgently needed to perform optical probing and take atomic sensors from proof-of-concept laboratory-based systems to portable and manufacturable devices.

One potential strategy to address the issue is to leverage the optical metasurfaces, which are two-dimensional (2D) arrays of nanostructures engineered at subwavelength and have emerged as a promising platform for integrated optical components with enhanced and augmented functionalities [12], [13], [14], [15], [16], [17], [18], [19]. In the last few years, there has been growing interest in the utilization of metasurfaces to perform atom trapping and manipulation. Both cold and hot atoms represent highly resourceful quantum technology platforms. As for the integration of cold atom quantum devices, the planar meta-optics have achieved important progress in revolutionizing the conventional magneto-optical trap (MOT) in a compact and robust fashion. Recent works have demonstrated diverse integration schemes towards metasurface-based atom trapping with the help of beam-splitter metasurface chip [20], [21], beam-shaping and polarization control metasurface [22], [23], metasurface-lens optical tweezer [24] and holographic metasurface optical trap arrays [25]. On the other hand, OPM, as a classic representative of quantum devices based on hot alkali atoms, has gradually developed towards chip-integration through meta-optics in the last two years. For example, polarization-multiplexed meta-devices have been designed to achieve optical rotation detection of probe light [26], [27], [28] or track the change of polarization induced by magnetically induced circular dichroism [29]. In addition, a waveplate-like metasurface designed by a computer-assisted optimization algorithm has been developed to achieve chip-scale OPM with elliptically polarized laser-pumped [30]. Despite these advances in the miniaturization of OPM, little attention has been focused on the integration of the optical probing system, which is essential for preparing initial polarized light to undergo subsequent optical rotation or absorption after interacting with hot alkali atoms. As for polarization conversion metasurface which is requisite in chip-scale OPM to conduct optical probing before light-atom interaction, although there has been some research in recent years on designing metasurfaces as polarizers, such as poincaré sphere polarizers [31], [32], [33], angle-multiplexed meta-waveplate [34], direction-controlled meta-polarizer [35] and multi-layer stacked complementary meta-polarizer [36], but combining them to facilitate the development of integrated atomic sensors has still not received much attention.

**Figure 1:**
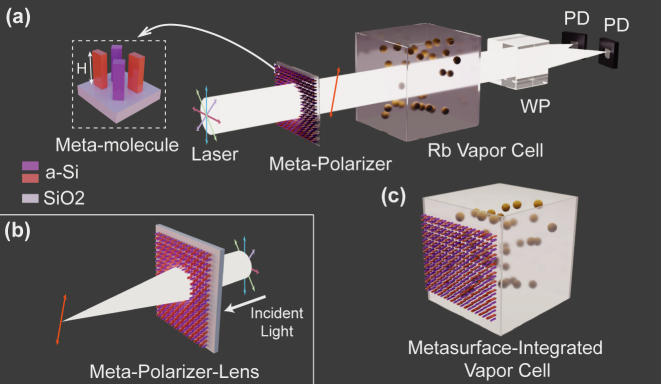
Chip-scale optically pumped magnetometer (OPM) concept and design. (a) Schematic diagram of the proposed integrated optical probing scheme enabled by localized-interference metasurface. WP, wollaston prism; PD, photodiode. (b) The proposed meta-polarizer-lens for tailorable linear polarization conversion and wavefront manipulation. (c) Conceptual sketch of the metasurface-integrated vapor cell that is interfaced directly with the proposed metasurface and other meta-optics.

In this paper, an integrated optical probing scheme leveraging localized-interference metasurface for chip-scale OPM is proposed, as shown in Figure 1(a). With the help of the extra design freedom provided by incorporating multiple meta-atoms in a single meta-molecule, localized interference at the subwavelength scale is realized with strong linear dichroism. The metasurface works as a tailorable linear meta-polarizer at the Rb D1 transition wavelength (*λ* = 795 nm), allowing the desired linear polarization state to be transmitted while blocking its orthogonal polarization component. By introducing the propagation phase, the meta-polarizer achieves full-space wavefront shaping and polarization conversion simultaneously inside the monolithic chip, which acts as the combination of a convex lens and a linear polarizer, i.e., meta-polarizer-lens, as shown in Figure 1(b). The meta-polarizer-lens plays a pivotal role in vertical-cavity surface-emitting (VCSEL)-based chip-scale OPM where both wavefront shaping and polarization conversion are urgently needed. The aforementioned optical probing scheme is experimentally characterized by employing the meta-polarizer as a plug-and-play component in the existing zero-field resonance OPM setup. A sensitivity of approximately 9 fT/Hz^1/2^ is achieved with the dynamic range of about ±2.3 nT after analyzing and suppressing the additional vector light shift induced by the meta-polarizer. The sensitivity based on our proposed integrated optical probing scheme is comparable to that of state-of-the-art conventional scheme utilizing cumbersome optical components [37]. Considering the complementary-metal-oxide-semiconductor (CMOS) compatibility of the metasurface manufacturing process, the vapor cell could be interfaced directly with the proposed metasurfaces and other meta-optics such as polarization splitter [38], [39], absorber [40] and reflector [41], as depicted in Figure 1(c).

## 2 Results and discussion

### 2.1 Theoretical design and simulation of the metasurface

The capability of metasurface is schematically shown in Figure 1(b), it can convert incident light with arbitrary polarization states into a desired linear polarization with a specific-designed wavefront. The unit cell is a localized-interference meta-molecule, which can be disassembled into two pairs of meta-atoms, each of them exhibits waveplate-like behavior with linearly birefringent properties and is capable of imparting distinct phases on orthogonal linear polarizations along its fast and slow axes [42]. As a proof of concept, the Jones matrix of the metamolecule could be defined as follows for arbitrary polarization to *x*-linear polarization conversion: (see Supplementary Note 1 for detailed derivation)

(1) $$\begin{array}{cc} J_{L} & =\left[\begin{array}{cc} 1 & 0\\ 0 & 0 \end{array}\right]\\ & =\frac{1}{2}R\left(-\frac{\pi }{4}\right)\left[\begin{array}{cc} 1 & 0\\ 0 & 1 \end{array}\right]R\left(\frac{\pi }{4}\right)+\frac{1}{2}\left[\begin{array}{cc} 1 & 0\\ 0 & -1 \end{array}\right] \end{array}$$

where the first term is physically realized by a linearly birefringent meta-atom with zero phase shifts along the fast and slow axes $\left(\left|\phi_{xx}^{1}|=|\phi_{yy}^{1}\right|\right)$ , complete transmission $\left(|t_{xx}^{1}|=|t_{yy}^{1}|=1\right)$ , and an orientation angle of −*π*/4; while the second meta-atom works as a half-waveplate (HWP) with phase shifts 0 and ±*π* along the fast and slow axes $\left(|\phi_{xx}^{1}|=|\phi_{xx}^{2}|=|\phi_{yy}^{2}|\pm \pi \right)$ , complete transmission $\left(|t_{xx}^{2}|=|t_{yy}^{2}|=1\right)$ as well as a zero orientation angle.

**Figure 2:**
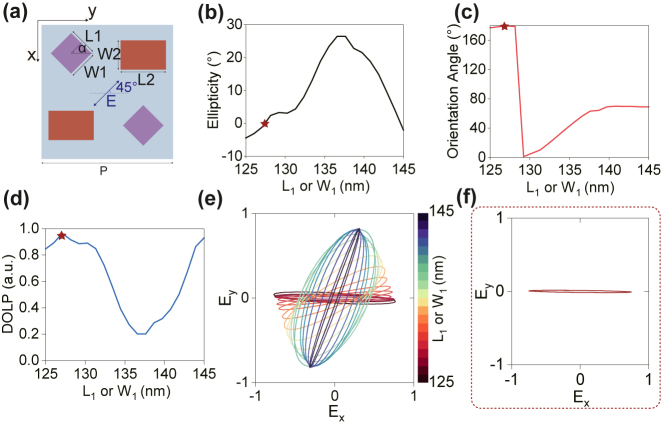
Optimal design of the meta-molecule to generate *x*-linear polarization. (a) Schematic of the metasurface unit cell. *P* = 800 nm. The simulation results of the (b) ellipticity *χ*, (c) orientation angle *ψ*, (d) degree of linear polarization (DOLP) and (e) polarization ellipse maps of transmitted light as a function of the side length (*L*_1_ or *W*_1_) of the first type meta-atom (meta-atom 1). (f) Optimal linear polarization generated by the best meta-molecule when *L*_1_ (or *W*_1_) = 127.5 nm.

**Figure 3:**
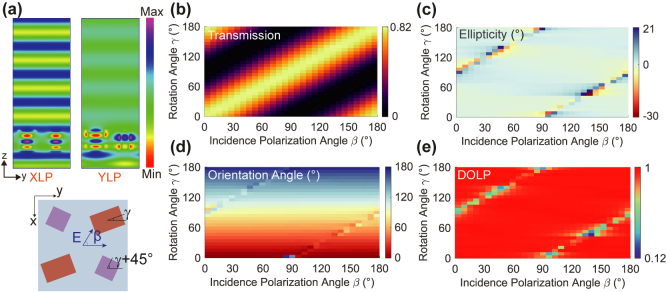
Validation of localized interference and performance characterization of the meta-polarizer for generating tailorable linear polarization states. (a) The electric field (*E*_*x*_) distribution within the optimal meta-molecule under the illumination of *x*- and *y*-linear polarizations at the designed wavelength of 795 nm. For the designed meta-polarizer with various rotation angle *γ* of HWP-like meta-atom 2 changing from 0° to 180°, the simulated (b) transmission, (c) ellipticity *χ*, (d) orientation angle *ψ*, and (e) DOLP as a function of incidence polarization angle *β*. The anomalous points in (c)–(e) correspond to the black region in (b).

The meta-atom is composed of amorphous silicon (a-Si) deposited on silica with period *P*_0_ = 400 nm and height *H* = 600 nm. More details about the simulation of the meta-atom can be found in Supplementary Note 2. Then, the meta-molecule is assembled by staggering two pairs of different meta-atoms at the center of each quadrant as depicted in Figure 2(a). The HWP-like meta-atom 2 ensures high polarization conversion, the length and width are *L*_2_ = 220 nm and *W*_2_ = 130 nm, respectively. In order to generate localized interference and enable the meta-molecule to act as a perfect linear polarizer, i.e., meta-polarizer, the polarization properties of transmitted light as a function of the length *L*_1_ or width *W*_1_ of the meta-atom 1 with a square cross-section (*L*_1_ = *W*_1_) and an orientation angle *α* = 45° are simulated. For the linearly incident light with a polarization angle of 45°, the ellipticity (*χ*), orientation angle (*ψ*) and degree of linear polarization (DOLP) of transmitted light are shown in Figure 2(b)–(d), respectively. DOLP, which defined as DOLP = 1 − 2|tan*χ*|/(1 + tan^2^ *χ*), represents linear polarization purity. As indicated by the red stars, when *L*_1_ (or *W*_1_) is equal to 127.5 nm, the meta-molecule exhibits strong linear dichroism and the simulated *χ*, *ψ* and DOLP of transmitted light are −0.3°, 178.8° and 0.96, respectively. All of them are quite close to the theoretical values of 0°, 180°, and 1. To display the variation process of the generated polarization states, the polarization ellipse maps associated with different *L*_1_ (or *W*_1_) are plotted in Figure 2(e), with the optimal case featured in Figure 2(f). The electric field (*E*_*x*_) distributions inside the optimal meta-molecule under the incidence of *x*- and *y*-linear polarizations are captured at *λ* = 795 nm, as shown in Figure 3(a). When designing with such meta-molecule structures, coupling between neighboring meta-atoms is not negligible, which potentially leads to perturbation of phase imparted by designed meta-atoms and deteriorates the device efficiency [43]. This coupling can be alleviated by strongly confining light within the meta-atoms or considering the coupling effects during the design process. In Figure 3(a), it is obvious that the electric fields are well confined inside the a-Si nanofins with high refractive index (≈3.614 at *λ* = 795 nm) and the near-field coupling is negligible. Therefore, the meta-atoms work as two independent birefringent waveplates and polarization-dependent interference occurs close to the meta-molecule’s exit surface, which agrees well with theory and indicates that the imparted phases of our designed meta-atoms are not affected by coupling or crosstalk effect. Such polarization-dependent interference results in transmission enhancement of the desired linear polarization while suppressing its orthogonal polarization.

In addition, exhaustive simulations are carried out to estimate the capability of tailorable linear polarization conversion. More details about theoretical analysis can be found in Supplementary Note 1. Figure 3(b)–(e) are the simulation results about transmission, *χ*, *ψ* and DOLP as a function of the incidence polarization angle *β* for various rotation angle *γ* of the HWP-like meta-atom 2. As shown in Figure 3(b), for a specific rotation angle *γ*, i.e., polarizer axis angle, the total transmission is in accordance with Malus’s law, which is defined as *I*_trans_ ∝ cos^2^(*β*). The transmission reaches maximum or minimum when the *β* and *γ* are equal or differ by 90°, respectively, which is consistent with that of an ideal linear polarizer. Figure 3(c)–(e) show the polarization properties of transmitted light. It is worth noting that the *χ*, *ψ* and DOLP nearly keep as 0, *γ* and 1 throughout the variation of *β* as expected unless in some anomalous cases where *β* ≈ *γ* ± 90°. The appearance of these anomalies can be attributed to the following reasons: when the incident polarization angle *β* is nearly perpendicular to the slow axis of the HWP-like meta-atom, the incident polarization is orthogonal to the desired linear polarization, implying that due to the destructive interference, almost no incident light can pass through the meta-molecule, as illustrated by the black region in Figure 3(b). As a result, minor fluctuations of transmitted light amplitude and phase are greatly exaggerated, with a high potential to generate elliptical polarization that deviates from expectation. By employing the multi-layer meta-molecule architecture, the deterioration of linear polarization can be mitigated [44].

**Figure 4:**
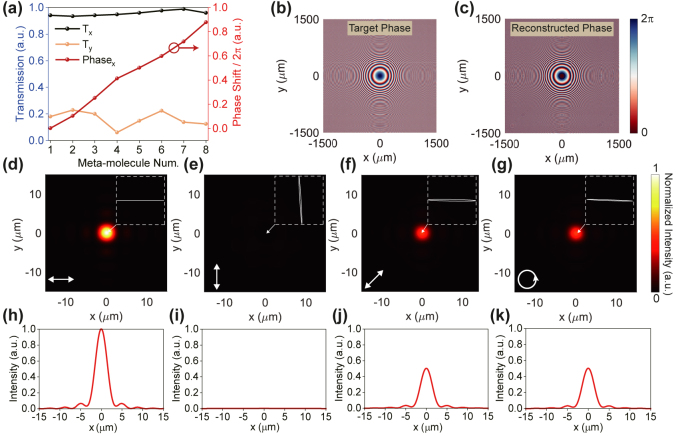
Characterization of the meta-polarizer-lens for wavefront manipulation of desired linear polarization. (a) Simulated transmission for *x*- and *y*-pol incident light and phase shift of eight selected meta-molecules with gradient phase delays of *π*/4 at *λ* = 795 nm. (b) Target and (c) reconstructed phase generated by the meta-polarizer-lens. (d)–(g) Simulated intensity profiles at the focal plane under *x*-, *y*-, 45°-pol and left-handed circular polarization (LCP) incidence. White square regions depict the polarization ellipse relating to each focal spot region. (h)–(k) Normalized intensity distributions along the *x*-axis at the focal plane corresponding to (d)–(g).

Moreover, the proposed metasurface could generate 0–2*π* phase profiles while exhibiting strong linear dichroism by combining the localized interference and propagation phase, allowing it to achieve linear polarization conversion and wavefront shaping simultaneously. Specifically, by carefully selecting the assembled meta-atoms, it is straightforward to obtain a series of meta-molecules arranged in a gradient phase satisfying 0–2*π* phase modulation. More details about theoretical analysis can be found in Supplementary Note 1. As shown in Figure 4(a), eight meta-molecules are designed to satisfy 2*π* phase coverage, each of them exhibits strong linear dichroism and can be treated as a linear meta-polarizer with a *π*/4 phase difference between each one. More details about the selected eight meta-molecules are shown in Supplementary Note 3. The multi-functional capacity of the designed metasurface is subsequently demonstrated through the meta-polarizer-lens, which can be regarded as an integration of a linear polarizer with a polarizer axis along the *x*-axis and a convex lens. The target focusing phase profile is shown in Figure 4(b), where the focal length of the meta-polarizer-lens is about 30 mm, corresponding to a numerical aperture (NA) [45] of about 0.05. Figure 4(c) depicts the reconstructed phase generated by the meta-polarizer-lens with a side length of 3 mm, which is nearly in line with the target phase. To conserve computing resources, a tiny metasurface with a side length of 30 μm and an NA of 0.1 is simulated. Figure 4(d)–(g) show the transmitted intensity distributions under different incident polarizations. The focusing efficiencies for *x*-pol, *y*-pol, 45°-pol, and left-handed circular polarization (LCP) are 79.76 %, 0.02 %, 40.45 %, 40.54 %, respectively. It is clear that the transmission is determined by the projection of the incident polarization states on *x*-linear polarization. In addition, the simulated polarization ellipses of the focused beam are drawn in white dashed regions, providing additional insight into the multi-functionality of the meta-polarizer-lens. On the one hand, as a convex lens, the meta-polarizer-lens achieves wavefront shaping as illustrated in Figure 4(h)–(k) with a focusing efficiency of up to about 80 %. On the other hand, as a linear polarizer, it delivers the output polarization with high DOLP. Such a multi-functional property is unattainable with conventional optical components.

### 2.2 Optical characterization of the localized-interference metasurface

**Figure 5:**
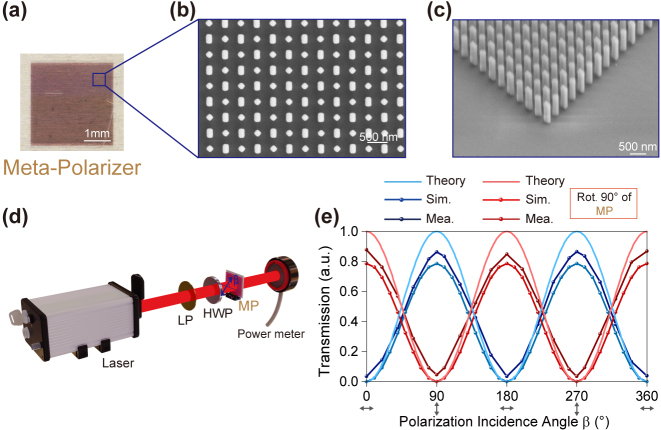
Optical characterization of the meta-polarizer. (a) Optical microscopy and (b)–(c) scanning electron microscopy (SEM) images of the fabricated meta-polarizer. (d) Schematic of the experimental setup for meta-polarizer characterization. *β* is the polarization incidence angle. LP, linear polarizer; HWP, half-waveplate; MP, meta-polarizer. (e) Theoretical, simulated and measured transmission of MP and rotation of MP by 90° under different incidence polarization angle *β*.

**Figure 6:**
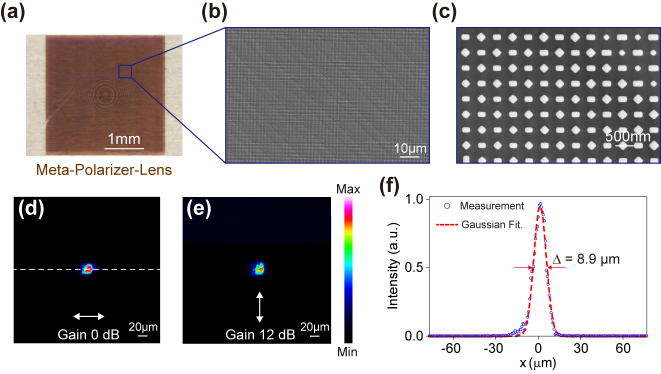
Optical characterization of the meta-polarizer-lens. (a) Optical microscopy and (b)–(c) SEM images of the fabricated meta-polarizer-lens. The measured intensity distributions at the focal plane under (d) *x*-pol (Gain 0 dB) and (e) *y*-pol (Gain 12 dB, about 4 times). Scalar bar: 20 μm. (f) Measured intensity distribution in the focal plane along the dotted line in (d), showing about 8.9 μm full-width-half-maximum (FWHM) of the focal spot.

To experimentally validate our proposed design, two localized-interference metasurfaces (meta-polarizer and meta-polarizer-lens), both with a large dimension of 3 × 3 mm^2^, are manufactured through a standard nanofabrication process, details about the fabrication are available in the **Methods** section. The optical microscopy and scanning electron microscopy (SEM) images of the meta-polarizer are shown in Figure 5(a)–(c). The meta-polarizer is characterized by an optical system as shown in Figure 5(d), where linearly polarized illumination with an arbitrary polarization angle can be realized by rotating the HWP. A commercial power meter is utilized to measure the intensity of the transmitted light. Taking a substrate region with the same area but lacking the metasurface pattern as a reference, the intensity results are further normalized. Figure 5(e) shows the transmitted intensity, which varies as a function of the polarization incidence angle *β*, where the results indicated by the red lines are obtained by rotating 90° of the meta-polarizer, demonstrating its ability to generate linear polarization with a tailorable polarization angle. The measured maximum and minimum transmission efficiencies are *T*_min_ = 3.24 % and *T*_max_ = 86.29 % at a polarization incidence angle of 0° and 90°, respectively, resulting in a high extinction ratio ER = 10log(*T*_max_/*T*_min_) = 14.2 dB. Regarding the rotating 90° of the meta-polarizer, the corresponding measured results are 3.57 %, 87.91 % and 13.9 dB, respectively. As shown in Figure 5(e), the corresponding simulation results of the meta-polarizer are also provided, the maximum and minimum transmission efficiencies are 78.637 % and 0.015 %, respectively, resulting in an ER of about 37.2 dB, which shows the potential of our design to achieve high-performance ER. It is worth noting that the measured non-zero minimum intensity value might probably be attributed to imperfections of meta-atoms during the manufacturing process. For example, due to the attachment and accumulation effects of reactive ions during the reactive ion etching (RIE) process, the etched a-Si nanofin ends up with a tapered sidewall profile [46], which could introduce additional phase retardation, causing undesired scattering or interference and deteriorating the device performance. In addition, potential over-etching leads to rounded edges and corners of the fabricated meta-atoms, such deformation also imposes undesired phase modulation and deteriorates the performance [44], [47]. Therefore, in the future, it will be important to carefully adjust and optimize the gas species, flow rates, bias power, and etching time during the RIE process to correct a sidewall profile as right-angled and fabricate sharp corners and edges. In addition, the meta-polarizer-lens is also fabricated through a similar process and the optical microscopy and SEM images are shown in Figure 6(a)–(c). The experimental setup for optical characterization is similar to the configuration shown in Figure 5(b), to determine the focusing performance, a CMOS camera beam profiler replaces the power meter to capture the transmitted light field, and the experimental results of the intensity profiles at the focal plane (*z* ≈ 28 mm) under *x*- and *y*-pol incidences are shown in Figure 6(d) and (e), respectively. As shown in Figure 6(d), under *x*-pol incidence, there is a distinct focal point that satisfies the constructive interference and the focusing efficiency is about 72.79 %, corresponding to the simulated results in Figure 4(d). In contrast, when the incident light switches to *y*-pol, the transmitted intensity decreases significantly with a focusing efficiency of about 16.72 %, as shown in Figure 6(e), indicating that the meta-polarizer-lens exhibits linear dichroism with ER of about 6.4 dB, which is smaller than the meta-polarizer. This is mainly because the linear dichroism of the meta-molecules constructing meta-polarizer-lens is not as strong as that of the meta-molecules for meta-polarizer. Figure 6(f) describes the intensity distribution along a horizontal line passing through the center of the focal spot shown in Figure 6(d). The full-width-half-maximum (FWHM) is approximately 8.9 μm, which closely fits with the diffraction-limited value of 7.95 μm (*λ*/2NA) [48]. The discrepancy could potentially come from the fabrication process and the optical system for focal spot capture. In addition to focusing, our proposed metasurface enables arbitrary phase modulation and can be exquisitely designed according to specific requirements.

### 2.3 Characterization of the integrated optical probing scheme in meta-polarizer-based OPM

**Figure 7:**
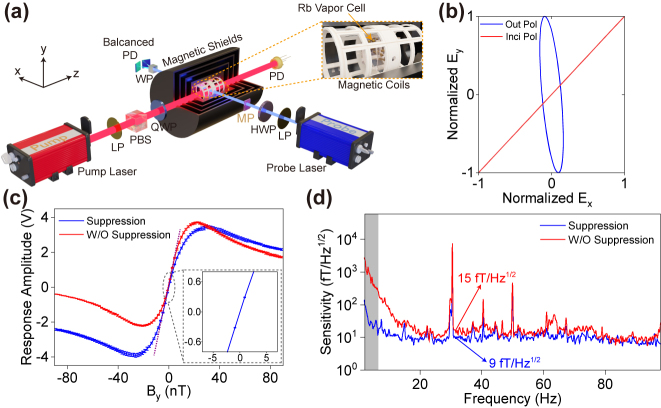
Experimental characterization of the integrated optical probing scheme in meta-polarizer-based OPM. (a) Sketch of the OPM experimental setup in which the meta-polarizer is placed in front of the glass vapor cell. LP, linear polarizer; HWP, half-waveplate; QWP, quarter-waveplate; PBS, polarization beam splitter; WP, wollaston prism; PD, photodiode; MP, meta-polarizer. (b) Measured normalized polarization ellipse of the incident light and transmitted light converted by the MP. (c) Meta-polarizer-based OPM output signal and (d) sensitivity before and after the vector light shift suppression.

The integrated optical probing scheme based on the meta-polarizer is carried out. As a proof of concept, a 4 × 4 × 4 mm^3^ vapor cell is combined with the meta-polarizer to construct a compact zero-field resonance OPM. Additional information regarding the experimental setup for magnetic field measurements can be found in **Methods** section. A schematic diagram of the experimental setup is shown in Figure 7(a), wherein the probe laser’s polarization is converted by the meta-polarizer, and then the probe laser interacts with the hot Rb atoms operating in the spin-exchange-relaxation-free (SERF) regime [49], where the spin-exchange rate is sufficiently larger compared to the atomic Larmor precession frequency. In this condition, the equilibrium state of atomic spin can be well characterized by a spin-temperature distribution and the electron spin evolution of alkali atom can be described by phenomenological Bloch equation:

(2) $$\frac{d}{dt}P=\gamma_{e}B\times P+\frac{1}{q}\left[R_{op}\left(s\hat{z}-P\right)-R_{\text{rel}}P\right]+D\nabla^{2}P$$

where $P={\left[P_{x},P_{y},P_{z}\right]}^{T}=\left\langle S\right\rangle /S$ is the electron spin polarization vector, **S** and *S* are the electron spin operator and the electron spin quantum number, respectively. *γ*_*e*_ is the gyromagnetic ratio of electron spin, and $B={\left[B_{x},B_{y},B_{z}\right]}^{T}$ is the applied magnetic field. *q* is the nuclear slow-down factor, which depends on the polarization of the alkali ensemble. The second items characterize the effect of various coherent interactions (such as wall collision, spin-destruction collision, etc.) at the relaxation rate *R*_rel_ and optical pumping interaction at pumping rate *R*_op_. *s* is the mean photon spin vector oriented in parallel with the direction of the pump beam. The last term describes the diffusion phenomenon of electron spin in the vapor cell, *D* is the diffusion coefficient. In our study, the diffusion term will be neglected because the high buffer gas pressure suppresses the diffusive motion of atoms. The polarization plane of linearly polarized probe light for optical probing undergoes rotation from interaction with the polarized alkali metal ensemble under the influence of magnetic-induced circular birefringence. This is known as the paramagnetic Faraday rotation, which is proportional to *P*_*x*_ and can be described as [50]:

(3) $$\theta =\frac{1}{2}r_{e}cf_{D1}nlP_{x}\frac{\nu_{pr}-\nu_{D1}}{{\left(\nu_{pr}-\nu_{D1}\right)}^{2}+{\left(\Gamma_{D1}/2\right)}^{2}}$$

where *r*_*e*_ is the classical electron radius, *l* is the length of light-atom interaction, *n* is the atomic density, *c* is the speed of light in vacuum, *ν*_pr_ is the frequency of the probe light, *f*_*D*1_ is the oscillator strength of Rb D1 line, *ν*_*D*1_ is the resonant frequency of the Rb D1 line modified by pressure-induced frequency shift, and Γ_*D*1_ is the pressure-broadened absorption linewidth of Rb vapor cell. The output signal of OPM can be obtained by measuring the optical rotation through polarimetry. When the residual magnetic field is zero, *P*_*x*_ is sensitive to the magnetic field along the *y*-axis, and the steady-state solution of is given by:

(4) $$P_{x}=P_{0}\frac{\left(R_{op}+R_{\text{rel}}\right)\gamma_{e}B_{y}}{{\left(R_{op}+R_{\text{rel}}\right)}^{2}+{\left(\gamma_{e}B_{y}\right)}^{2}}$$

where *P*_0_ is the equilibrium electron spin polarization. More details about the working principle of the SERF OPM are presented in the Supplementary Note 4.

In our experiment, the meta-polarizer converts linear polarization for optical probing. As shown in Figure 7(a), a conventional linear polarizer (LP) and a half-waveplate (HWP) are utilized to change the incident polarization before the meta-polarizer. By adjusting the polarization of light incident on the meta-polarizer to 45° linear polarization, the polarization of transmitted light is near *y*-pol with the orientation angle and ellipticity of about 95.42° and 7.43°, respectively, as shown in Figure 7(b). Such ellipticity primarily results from the non-infinite ER of the meta-polarizer. When optical probing is performed using elliptically polarized light, the presence of light shift will deteriorate the OPM’s response and performance [51]. Thus, it is crucial to thoroughly analyze and suppress the light shift caused by the non-ideal meta-polarizer to achieve high-performance chip-scale OPM. In SERF OPM, only the vector light shift should be considered and is typically expressed as follows: (more in-depth analysis and derivation could be found in Supplementary Note 5)

(5) $$L_{\text{vector}}=-\frac{I}{h\nu_{p}}\frac{r_{e}f_{D1}c}{\gamma_{e}}\frac{\nu_{pr}-\nu_{D1}}{{\left(\nu_{pr}-\nu_{D1}\right)}^{2}+{\left(\Gamma_{D1}/2\right)}^{2}}A$$

where *I* is the probe laser intensity, *hν*_*p*_ denotes the energy of a single photon. A is the degree of circular polarization (DOCP) of light for optical probing. Here, A=0 for the linearly polarized light, A=±1 for the right-handed and left-handed circularly polarized light, respectively. The vector light shift imitates the behavior of a magnetic field [52]. It could be suppressed through some methods presented by our group such as DOCP regulation scheme [53] or *in-situ* precise magnetic compensation [54]. Considering that the first regulation scheme requires an additional quarter-waveplate (QWP) to compensate for the undesired DOCP of probe laser induced by the non-ideal meta-polarizer, whereas *in-situ* precise magnetic compensation only needs to finely tune the driven current of magnetic coils according to the response of meta-polarizer-based OPM. To make the OPM system leaner and more compact, during the experiment, *in situ* magnetic field compensation is utilized to suppress the unwanted light shift, and the typical output signals with the dispersive shape of the compact OPM before and after the vector light shift suppression are shown in Figure 7(c). The existence of vector light shift is synonymous with a bias magnetic field along the probe beam axis, leading to an asymmetric and diminished output signal [54]. The detected signal after light shift suppression depends linearly on the magnetic field in the range of approximately ±2.3 nT, i.e., the dynamic range of the OPM. Furthermore, the presence of the vector light shift causes the transverse field to diverge from zero and results in magnetic-optical coupling effects, which deteriorate the sensitivity of OPM, particularly at low frequencies [51], as depicted by the shaded region in Figure 7(d). Considering that the SERF OPMs are mainly employed for detecting static or quasi-static magnetic fields, it is necessary to improve the low-frequency sensitivity by suppressing the vector light shift. The sensitivity of the OPM is determined by the noise floor, which is the lowest point near the frequency of the calibrated magnetic field (30.5 Hz in our study). The sensitivities are about 15 fT/Hz^1/2^ and 9 fT/Hz^1/2^ before and after the vector light shift suppression, respectively. Therefore, the performance of the meta-polarizer, especially ER, should be improved to enhance the sensitivity of chip-scale OPM by further optimizing the lithography and etching process or by employing advanced design strategies, such as topology optimization [55] or inverse design [56], [57], [58].

## 3 Conclusion

In summary, an integrated optical probing scheme enabled by localized-interference metasurface for chip-scale OPM is proposed. The designed metasurface comprises two pairs of asymmetrically birefringent meta-atoms with distinct intersection angles, allowing us to exquisitely configure their localized interference for ideal linear polarization conversion. Through exhaustive simulation and experimental characterization, the meta-polarizer with a maximum transmission efficiency of 86.29 % and a measured ER of approximately 14.2 dB at *λ* = 795 nm is presented. The polarization angle of the meta-polarizer can be adjusted by rotating the meta-molecules. Furthermore, the localized interference is combined with the propagation phase to construct the meta-polarizer-lens, which serves as the combination of a conventional convex lens and a linear polarizer with a focusing efficiency of 72.79 % and an ER of about 6.4 dB. As a proof of concept, the feasibility and practicality of the integrated optical probing scheme are characterized by combining the meta-polarizer and a miniaturized vapor cell to build a compact zero-field resonance OPM. High sensitivity up to about 9 fT/Hz^1/2^ is achieved after suppressing the vector light shift induced by the non-ideal meta-polarizer, which is comparable to that of state-of-the-art conventional optical probing scheme [37], confirming the feasibility of the proposed integrated approach. Recently, VCSELs have gradually been applied to promote the development of miniaturized and compact OPM [59], compared with the widely used fiber-coupled distributed feedback (DFB) lasers, VCSELs offer unique advantages such as on-wafer characterization, small size and low consumption. In the future, the meta-polarizer-lens will play a significant role in the VCSEL-based chip integration of OPM, where wavefront shaping and polarization conversion are urgently demanded [59]. Moreover, the unique planar configuration, high throughput and CMOS-compatibility fabrication of metasurfaces make them promising candidates not only for integration on the surface of microfabricated vapor cell, but also for optoelectronic monolithic integration with VCSEL [60]. In the future, we will focus on the integration of VCSEL, meta-optics, and microfabricated vapor cell to realize metasurface-integrated VCSEL and vapor cell to advance the development of chip-scale OPM. We envision that our work extends the applicability of meta-optics by providing integrated optical probing solutions for emerging atomic sensors, and promoting the practicality and scalability of atomic devices beyond what is possible with conventional and discrete optical components.

## 4 Methods

### 4.1 Simulation

More details about the meta-atom design and simulation can be found in Supplementary Note 2.

### 4.2 Fabrication

First, a thin amorphous silicon (a-Si) layer with a ∼600 nm thickness was deposited onto a silica substrate via plasma-enhanced chemical vapor deposition (PECVD). Then, the optical properties of the film were characterized and the wavelength-dependent refractive index was measured with spectroscopic ellipsometry, more details about the characterized results are shown in the Supplementary Note 6. Subsequently, electron-beam lithography (Elionix, ELS-BODEN 125) was utilized to obtain the patterned a-Si metasurface utilizing a negative tone resist (All Resist, AR7520.17). The pattern was transferred onto the a-Si layer via inductively coupled plasma-reactive ion etching (ICP-RIE), with AR7520.17 negative tone resist serving as the mask directly. Last, the AR7520.17 resist was stripped by the remover (All Resist, AR 300-73).

### 4.3 Experimental setup of meta-polarizer-based OPM

A 4 × 4 × 4 mm^3^ micro-miniature borosilicate cubic alkali vapor cell containing a droplet of ^87^Rb metal and 450 Torr of N_2_ buffer gas was fabricated for our experiment. Considering the pressure-induced frequency shift, the center wavelength of the ^87^Rb D1 transition is 795.0095 nm. The Rb atom was heated to 150 °C through twisted-pair wires with AC currents driving and an Rb density of about 10^14^ cm^−3^ is achieved. This hot vapor cell was placed at the center of magnetic shields formed by a four-layer cylindrical μ-metal and a single-layer ferrite to shield the earth’s magnetic field and suppress the magnetic noise. The residual magnetic field inside the shield was further compensated by a set of triaxial magnetic coils, which are composed of Lee–Whiting coils and saddle coils with a uniform field around the area of the vapor cell. The magnetic coils were driven by function generators (3350B, Keysight). The pump and probe lasers were generated by two distributed feedback (DFB) diode lasers dedicated to quantum sensing (Toptica, DL pro and DFB pro). The pump laser was tuned to the resonant frequency of the D1 line and was impinged on the vapor cell after being converted into a circular polarization via a combination of LP, PBS, and QWP. The probe laser was about 50 GHz far detuned from the center of the D1 line to reduce the light absorption and maximize the OPM signal. The polarization of the probe laser was regulated by a polarization system consisting of an LP, HWP and the meta-polarizer. The meta-polarizer was mounted on a three-axis motorized stage (Thorlabs, MT3/M) for precise position adjustment. The polarization of laser transmitted through the meta-polarizer was monitored by commercial polarimetry (Thorlabs, PAX1000IR1/M). The polarization evolution of the probe laser after interacting with alkali vapors was tracked by a balanced polarimetry made up of a Wollaston prism and a pair of photodiodes. The photo-current output by the balanced PD, i.e., the output signal of OPM, was amplified and changed to a voltage signal by a transimpedance current amplifier (TIA) and then collected by a data acquisition (DAQ) system.

## Supplementary Material

Supplementary Material Details
