# Supplementary material for: Integrated optical probing scheme enabled by localized-interference metasurface for chip-scale atomic magnetometer
Source: Nanophotonics. 2024 Sep 26;13(23):4231–42. doi: 10.1515/nanoph-2024-0296 (PMC11636512; doi:10.1515/nanoph-2024-0296)
Supplement: Supplementary file 1 — Supplementary Material Details [file j_nanoph-2024-0296_suppl_001.pdf]

Supplementary Material for

Integrated Optical Probing Scheme Enabled by Localized-Interference Metasurface for Chip-Scale Atomic Magnetometer

Jinsheng Hu[a,b], Zihua Liang[a,b], Peng Zhou[a,b], Lu Liu[a,b], Gen Hu[a,b], Mao Ye[a,b,c,d,*]
[a]Key Laboratory of Ultra-Weak Magnetic Field Measurement Technology, Ministry of Education, School of Instrumentation and Optoelectronic Engineering, Beihang University, Beijing 100191, China
[b]Zhejiang Provincial Key Laboratory of Ultra-Weak Magnetic-Field Space and Applied Technology, Hangzhou Innovation Institute, Beihang University, Hangzhou 310051, China
[c]Hangzhou Institute of Extremely-Weak Magnetic Field Major National Science and Technology Infrastructure, Hangzhou 310051, China
[d]Hefei National Laboratory, Hefei 230088, China

* Corresponding Author: Mao Ye, maoye@buaa.edu.cn

Supplementary Note 1. Jones matrix analysis of the proposed localized-interference metasurface

The working mechanism of the waveplate-like meta-atom utilized in our study can be analyzed by the sophisticated Jones matrix:

$$\mathbf{J_0} = \mathbf{R}(-\psi_0)\begin{bmatrix} |t_{xx}|e^{i\phi_{xx}} & 0 \\ 0 & |t_{yy}|e^{i\phi_{yy}} \end{bmatrix}\mathbf{R}(\psi_0),\ \ \mathbf{R}(\psi_0) = \begin{bmatrix} \cos\psi_0 & -\sin\psi_0 \\ \sin\psi_0 & \cos\psi_0 \end{bmatrix} \tag{S1}$$

where $|t_{xx}|$ and $|t_{yy}|$ represent the transmission amplitudes for light polarized along the $x-$ and $y-$axes, respectively, and ϕ_{xx} and ϕ_{yy} represent the phase shifts along the $x-$ and $y-$axes. $\mathbf{R}$ is rotation matrix. It is evident that a metasurface composed of only one type of these linearly birefringent meta-atoms acts as a distinct optical element depending on the polarization of incident light, which implies that it is impossible to achieve arbitrary to fixed polarization conversion. Here, we will explore how the unit cell composed of meta-molecules containing two different types of linearly birefringent meta-atoms can transform arbitrarily polarized light to specified linear polarization and achieve wavefront control simultaneously. In the global linear polarization base defined in the xoy coordinate system, the incident and transmitted light amplitude vector ($\mathbf{i_L}$, $\mathbf{t_L}$) with arbitrary polarization could be expressed as the combination of the linear components on the x-axis and the y-axis.

The general Jones matrix $\mathbf{J}$, which characterizes the meta-molecule, relates the complex amplitudes of the incident light $\mathbf{i_L} = [i_x, i_y]^T$ and transmitted light $\mathbf{t_L} = [t_x, t_y]^T$ in the xoy coordinate as follows:

$$\mathbf{t_L} = \mathbf{J}\mathbf{i_L} \rightarrow \begin{bmatrix} t_x \\ t_y \end{bmatrix} = \begin{bmatrix} T_{xx} & T_{xy} \\ T_{yx} & T_{yy} \end{bmatrix}\begin{bmatrix} i_x \\ i_y \end{bmatrix}, \tag{S2}$$

Where the $T_{ab}(a, b \in x, y)$ represents the transmission coefficient of a component electric field under b component electric field incidence. As we consider the planar unit cell proposed in this paper with reflection symmetry in the propagation direction (xoz or yoz plane), the transmission coefficient of the linear cross-polarization components are equal, i.e., $T_{xy} = T_{yx}$, so the Jones matrix $\mathbf{J}$ is a symmetric matrix which could be described as follows:[1,2]

$$\mathbf{J} = \begin{bmatrix} T_{xx} & T_{xy} \\ T_{xy} & T_{yy} \end{bmatrix} = \begin{bmatrix} a & b \\ b & c \end{bmatrix} \tag{S3}$$

Firstly, we take the polarization conversion of $x-$pol as the instance to demonstrate, we find that the perfect linear polarization conversion in Equation S3 requires $a = 1$ and $b = c = 0$:

$$\mathbf{J_L} = \begin{bmatrix} 1 & 0 \\ 0 & 0 \end{bmatrix} = \frac{1}{2}\mathbf{R}(-\frac{\pi}{4}) \begin{bmatrix} 1 & 0 \\ 0 & 1 \end{bmatrix} \mathbf{R}(\frac{\pi}{4}) + \frac{1}{2} \begin{bmatrix} 1 & 0 \\ 0 & -1 \end{bmatrix} \tag{S4}$$

which is the same as Equation 1 in the main text. In addition, tailorable linear polarization state generation could be achieved by employing an additional rotation angle γ, the new Jones matrix of Equation S4 can be described as follows:

$$\mathbf{J^r} = \mathbf{R}(-\gamma) \begin{bmatrix} 1 & 0 \\ 0 & 0 \end{bmatrix} \mathbf{R}(\gamma) = \begin{bmatrix} \cos^2\gamma & \cos\gamma\sin\gamma \\ \sin\gamma\cos\gamma & \sin^2\gamma \end{bmatrix} \tag{S5}$$

An arbitrary elliptical polarization state $\mathbf{a}$ can be completely expressed by its orientation angle ψ and ellipticity χ, which can be described by a particular coordinate (2ψ, 2χ) on a Poincaré sphere.[3,4] In the global xoy linear polarization base, the Jones vector of $\mathbf{a}$ can be represented as follows:

$$\mathbf{a} = \mathbf{R}(\psi) \begin{bmatrix} \cos\chi \\ -i\sin\chi \end{bmatrix} = \begin{bmatrix} \cos\psi\cos\chi + i\sin\psi\sin\chi \\ \sin\psi\cos\chi - i\cos\psi\sin\chi \end{bmatrix} \tag{S6}$$

The transmitted light $\mathbf{a^L_{out}}$ after passing through the meta-molecule with an addition rotation angle γ could be described by:

$$\mathbf{a^L_{out}} = [\cos\gamma(\cos\psi\cos\chi + i\sin\psi\sin\chi) + \sin\gamma(\sin\psi\cos\chi - i\cos\psi\sin\chi)] \begin{bmatrix} \cos\gamma \\ \sin\gamma \end{bmatrix} \tag{S7}$$

The obtained Equation S7 demonstrates that the resulting meta-molecule is analogous to a linear polarizer with a polarizer axis angle equal to the additional rotation angle γ. By elaborately modifying the size of each meta-atom, the propagation phase will be altered exquisitely. While guaranteeing nearly complete transmission ($|t_{xx}^{1\_new}| = |t_{yy}^{1\_new}| = 1$), we assume that $\phi_{xx}^{1\_new}$ and $\phi_{yy}^{1\_new}$ represent the new phase shifts of the meta-atom 1 ($\phi_{xx}^{1\_new} = \phi_{yy}^{1\_new}$), in this case, to satisfy the condition of localized interference, new phase shifts of the HWP-like meta-atom 2 should satisfy $\phi_{xx}^{2\_new} = \phi_{xx}^{1\_new} = \phi_{yy}^{2\_new} \pm \pi$. After a few derivations, the Jones matrix of the meta-molecule

with both phase modulation and localized interference can be described as follows:

$$\mathbf{J}_{\mathbf{L}}^{\mathbf{new}} = e^{i\phi_m}\frac{1}{2}\mathbf{R}(-\frac{\pi}{4})\begin{bmatrix} 1 & 0 \\ 0 & 1 \end{bmatrix}\mathbf{R}(\frac{\pi}{4}) + e^{i\phi_m}\frac{1}{2}\begin{bmatrix} 1 & 0 \\ 0 & -1 \end{bmatrix} = e^{i\phi_m}\mathbf{J}_{\mathbf{L}} \tag{S8}$$

The transmitted light $\mathbf{a}_{\mathbf{out\_new}}^{\mathbf{L}}$ can be expressed as:

$$\mathbf{a}_{\mathbf{out\_new}}^{\mathbf{L}} = e^{i\phi_m}\mathbf{J}_{\mathbf{L}}\mathbf{a} = e^{i\phi_m}\mathbf{a}_{\mathbf{out}}^{\mathbf{L}} \tag{S9}$$

Where $\phi_m = \phi_{xx}^{1\_new} - \phi_{xx}^{1}$, Equation S9 indicates that the new meta-molecule after adjusting the size of each meta-atom can be designed to have the same polarization generation, while the transmitted light carries ϕ_m phase difference.

Supplementary Note 2. Meta-atom design and simulation

As shown in Figure S1a, each meta-atom is composed of a 600 nm tall (H = 600 nm) amorphous silicon (a-Si) nanofin, which is deposited on a silica substrate with a thickness of 500 μm. We simulate the transmission amplitude and phase of the meta-atom as a function of the length (L) and width (W) by means of the finite-difference time-domain (FDTD) method to build the parameters library. This simulation is performed for both $x-$ and $y-$linearly polarized incident light at the wavelength of 795 nm, corresponding to the D1 transition wavelength of Rb. Along $x-$ and $y-$axes, periodic boundary conditions are applied and perfectly matched layer boundary condition is utilized in the $z-$direction. The length L and width W of the meta-atom are swept within a range from 100 nm to 350 nm at an interval of 2.5 nm, with the fixed period P_0 = 400 nm, height H = 600 nm, and orientation angle ψ_0 = 0°. The simulation results for transmission (t_{xx}) and phase (ϕ_{xx}) of $x-$polarized incident light are depicted in Figure S1b and c, respectively. The transmission and phase of $y-$polarized incident light are the transposes of L and W of the response for $x-$polarized light. To realize independent and continuous phase control for both $x-$ and $y-$polarized light, the optimal cross-sectional parameters (L, W) of the meta-atom can be determined by minimizing the Euclidean distance method,[5] as shown in Figure S1d,e. The transmissions of selected meta-atoms for $x-$polarized (T_{xx}) and $y-$polarized (T_{yy}) corresponding to Figure S1d,e are mostly higher than 0.93, as shown in Figure S1f,g.

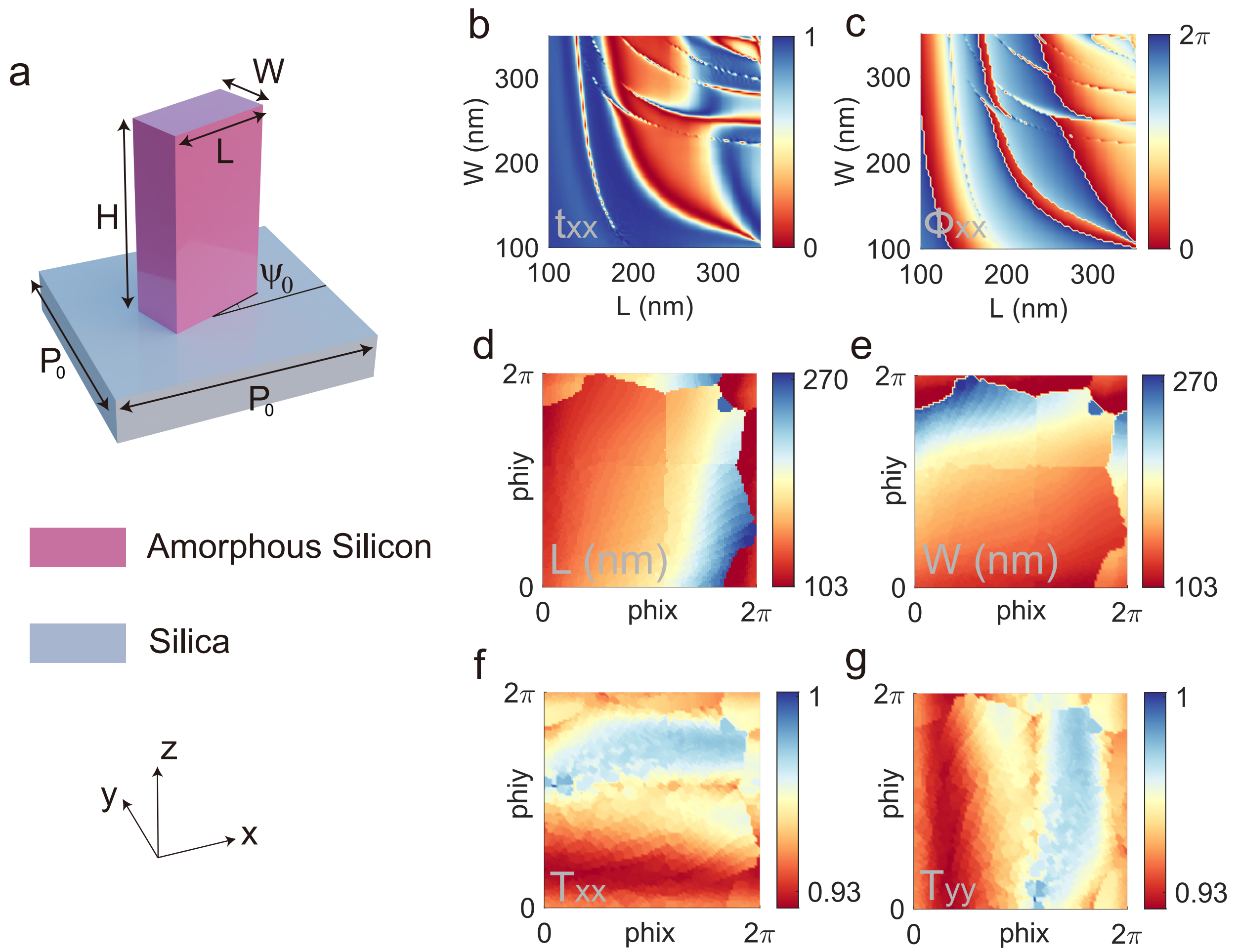

Figure S1: Meta-atom design and simulation. (a) Tilted view of the meta-atom. (b) Simulated transmission (t_{xx}) and (c) phase (ϕ_{xx}) for $x-$linearly polarized incident light when the rotation angle $\psi_0 = 0°$. (d) Selected length (L) and (e) width (W) of the meta-atom for independent and continuous control of the phase of $x-$ and $y-$linearly polarized light. (f) Simulated transmission for $x-$polarized (T_{xx}) and (g) $y-$polarized (T_{yy}) of selected meta-atoms to realize independent phase control.

Supplementary Note 3. Meta-molecule design of meta-polarizer-collimator for linear polarization generation and wavefront manipulation

Based on the propagation phase, it is demonstrated that the proposed localized-interference metasurface can achieve full-space phase manipulation (0-2π). As shown in Figure S2, a set of eight meta-molecules are chosen with a gradient phase satisfying 0-2π and the specific geometric parameters of these meta-molecules can be found in Table S1, where L_1 and W_1 relate to the assembled meta-atom 1 and L_2 and W_2 represent the length and width of HWP-like meta-atom 2. As depicted in Figure S2b and d, the co-polarization transmission (t_{xx} and t_{yy}) of meta-atom 1 and 2 exhibit a high degree of similarity (>0.95). It has been confirmed that the phase delays for $x-$ and $y-$polarized light of meta-atom 1 are almost equal in each meta-molecule. Besides, they follow a gradient phase distribution at an interval of $\pi/4$ among different meta-molecules, as shown in Figure S2c. Two pair of meta-atoms construct the meta-molecule with period P = $2P_0$ = 800 nm and they are considered as a whole to realize the manipulation of the wavefront, thus it is crucial that two meta-atoms in each meta-molecule have the same phase delay, i.e., ϕ_{xx} (meta-atom 1) = ϕ_{xx} (meta-atom 2), which is ensured in Figure S2e. Meanwhile, according to the Jones Matrix of the meta-molecule (Equation S8), the meta-atom 2 exhibits behavior similar to a half-waveplate (HWP), which implies that the phase delays difference between $x-$ and $y-$polarized incident light in each meta-molecule must be $\pm\pi$, which is also verified in Figure S2e.

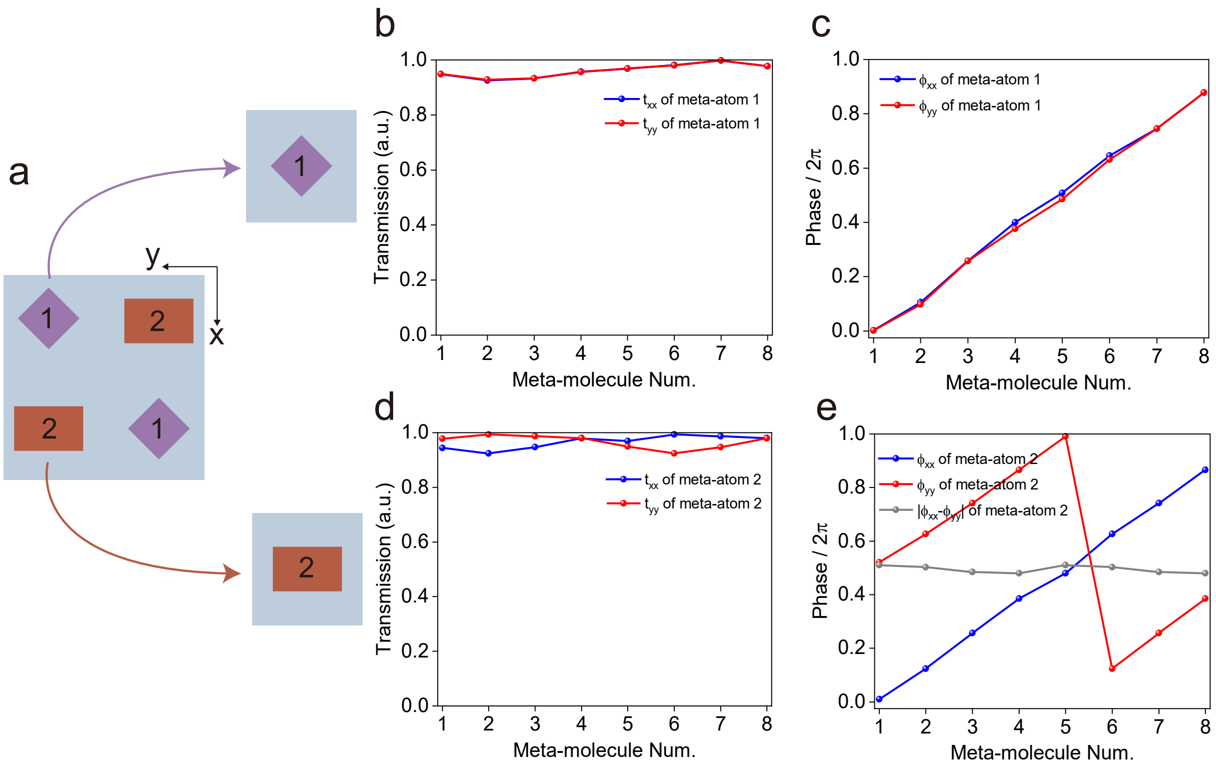

Figure S2: Meta-molecule design and simulation. (a) Schematic diagram of the meta-molecule consisting of two pairs of meta-atoms (meta-atom 1 and meta-atom 2). (b) Transmission and (c) Phase of each meta-atom 1 in selected meta-molecules. (d) Transmission and (e) Phase of each HWP-like meta-atom 2 in selected meta-molecules. For meta-atom 2, $|\phi_{xx} - \phi_{yy}| = \pi$ is almost satisfied in all selected meta-molecules.

Table S1: The geometric parameters of each meta-atom within eight selected meta-molecules for linear polarization generation and wavefront modulation (unit: nm)

Meta-Atoms	Parameters	M1	M2	M3	M4	M5	M6	M7	M8
1	$L_1 = W_1$	128	137	145	150	156	165	180	118
2	L_2	115	125	130	135	180	188	205	240
2	W_2	180	188	205	240	115	125	130	135

Supplementary Note 4. Working principle of dual-beam optically pumped magnetometers operating in the spin-exchange-relaxation-free (SERF) regime

High-sensitivity optically pumped magnetometers (OPMs) mainly rely on spin-polarized atomic vapors through optical pumping. OPM research closely relates to the atomic physics sub-field investigating spin interactions in such media. Within the Schrödinger picture, the evolution of alkali ensemble-averaged electron spin density matrix ρ for magnetic field interactions, relaxation due to spin-exchange and other collision processes, and optical pumping is described by the following density matrix equation:[6,7]

$$\begin{aligned}\frac{\partial \rho}{\partial t} =& \frac{1}{i\hbar}[a_{hf}\mathbf{I}\cdot\mathbf{S} + \gamma_e \hbar \mathbf{B}\cdot\mathbf{S}, \rho] + R_{se}[\varphi(1+4\langle\mathbf{S}\rangle\cdot\mathbf{S}) - \rho] + \\ &+ R_{rel}[\varphi - \rho] + R_{op}[\varphi(1+2\mathbf{s}\cdot\mathbf{S}) - \rho] + D\nabla^2\rho\end{aligned} \tag{S10}$$

where

$$\varphi = \frac{1}{4}\rho + \mathbf{S}\cdot\rho\mathbf{S} \tag{S11}$$

is the pure part of the density matrix. Here ρ has the dimension of the number of hyperfine states. The first term in Equation S10 is the ground state Hamiltonian with hyperfine coupling and Zeeman interaction with external magnetic fields. $\hbar$ is the Planck constant, and $\mathbf{S}$ and $\mathbf{I}$ are the electron spin operator and the nuclear spin operator in alkali atoms, respectively. a_{hf} is the hyperfine splitting constant, $\gamma_e = g_J\mu_B/\hbar$ is the gyromagnetic ratio of electron spin, and $\mathbf{B} = [B_x, B_y, B_z]^T$ is the applied magnetic field. The second item represents the evolution of ρ with alkali spin-exchange collision interactions under collision rates R_{se}. The distinction between $\langle\mathbf{S}\rangle$ and $\mathbf{S}$ is that the former is an expectation value, while the latter is an operator, i.e., $\langle\mathbf{S}\rangle$ = Tr(ρS). The third and fourth items characterize the effect of various coherent interactions (such as wall collision, spin-destruction collision, etc.) at the relaxation rate R_{rel} and optical pumping interaction at pumping rate R_{op}. $\mathbf{s}$ is the mean photon spin vector oriented in parallel with the direction of the pump beam and its magnitude depends on the degree of circular polarization (DOCP). The last term describes the diffusion phenomenon of electron spin in the vapor cell, D is the diffusion coefficient.

Among various OPMs, the so-called spin-exchange-relaxation-free (SERF) magnetometer introduced by the Princeton group has the highest sensitivity down to sub-fT/Hz$^{1/2}$ at low frequency.[8,9] The absence of relaxation from spin-exchange collisions (i.e., $R_{se} = 0$) is the key to the high sensitivity of the SERF magnetometer. This condition could be realized under high

densities of alkali vapor ($\sim 10^{14}$ cm^{-3}), resulting in the spin-exchange rate greatly exceeding other relaxation rates and optical pumping rate. In this case, the atomic population reaches equilibrium after many spin-exchange collisions, which is analogous to the thermal equilibrium described by the Boltzmann distribution, also known as the spin-temperature distribution. Additionally, at the near-zero magnetic field environment, the spin precession is sufficiently slow and a convenient simplification over the complicated density matrix can be modeled by the phenomenological Bloch equation:[7]

$$\frac{d}{dt}\mathbf{P} = \gamma_e \mathbf{B} \times \mathbf{P} + \frac{1}{q}[R_{op}(s\hat{z} - \mathbf{P}) - R_{rel}\mathbf{P}] + D\nabla^2\mathbf{P} \tag{S12}$$

where $\mathbf{P} = [P_x, P_y, P_z]^T = \langle \mathbf{S} \rangle / S$ is the electron spin polarization vector, $\mathbf{S}$ and S are the electron spin operator and the electron spin quantum number, respectively. q is the nuclear slow-down factor, which depends on the polarization of the alkali ensemble. In this paper, the probe beam with linear polarization and the pump beam with circular polarization are propagated along the x-axis and z-axis, respectively. The diffusion term will be neglected in our system because the high buffer gas pressure suppresses the diffusive motion of atoms. q is the nuclear slow-down factor, which depends on the polarization of the alkali ensemble. The second term in Equation S12 describes the effect of optical pumping to polarize the spin along the $z-$axis, while the third term characterizes the effect of spin relaxation to depolarize the spin polarization. In the case of a slowly varying magnetic field, the steady-state solution of the Bloch equation can be utilized to characterize the dynamic of spins and obtain the output signal of SERF OPM:

$$P_x = P_0 \frac{(R_{op} + R_{rel})\gamma_e B_y + \gamma_e^2 B_x B_z}{(R_{op} + R_{rel})^2 + \gamma_e^2 (B_x^2 + B_y^2 + B_z^2)} \tag{S13}$$

where $P_0 = \frac{R_{op}}{R_{op}+R_{rel}}$ is the equilibrium electron spin polarization. Off-resonant linearly polarized probe light is utilized to implement optical probing and detect the spin polarization of atom spins, the polarization axis of the probe light rotates as it interacts with the polarized atoms, also known as paramagnetic Faraday rotation. The magnetometer signal can be obtained by measuring the optical rotation of probe light through polarimetry,[10] and the optical rotation of the linearly polarized probe beam is proportional to P_x:

$$\theta = \frac{1}{2} r_e c f_{D1} n l P_x \frac{\nu_{pr} - \nu_{D1}}{(\nu_{pr} - \nu_{D1})^2 + (\Gamma_{D1}/2)^2} \tag{S14}$$

where r_e is the classical electron radius, l is the length of light-atom interaction, n is the atomic

density, c is the speed of light in vacuum, ν_{pr} is the frequency of the probe light, f_{D1} is the oscillator strength of Rb D1 line, ν_{D1} is the resonant frequency of the Rb D1 line modified by pressure-induced frequency shift, and Γ_{D1} is the pressure-broadened absorption linewidth of Rb vapor cell.

Supplementary Note 5. Analysis of light shift in OPM with dense hot alkali vapor and high-pressure buffer gas

In OPM with dense alkali vapor, off-resonant optical probing is recognized as an effective method for analyzing the dynamics of atomic ensembles in the presence of light and external magnetic fields. However, non-resonant interactions might also cause modifications of atomic states by AC Stark energy-level shifts, also known as light shifts.[11] For ground-state alkali-metal atoms such as Rb, there are numerous forms of light shifts: scalar light shifts (SLSs) of the net (or center of mass) ground-state energy or the hyperfine structure, vector light shifts (VLSs) of the Zeeman structure, and additional tensor light shifts (TLSs). Light shifts are quantified by means of atomic dynamic polarizability.[12] In our work, the polarizabilities of the ground 5s and excited 5p states (particularly for the D1 line of Rb) are determined. Due to the uncomplicated single-core electron structure of this atom, the angular momentum J and m_J values of the given atomic state are sufficient to describe the atomic polarizability α_v:[13]

$$\alpha_v(\omega) = \alpha_v^0(\omega) + \mathcal{A}\cos\theta_k \frac{m_J}{J}\alpha_v^1(\omega) + \left\{\frac{3\cos^2\theta_p - 1}{2}\right\}\frac{3m_J^2 - J(J+1)}{J(2J-1)}\alpha_v^2(\omega) + O^{(4)}, \quad \text{(S15)}$$

where $\mathcal{A}$, θ_k, θ_p define the degree of circular polarization (DOCP), the angle between the wave vector of the electric field and the direction of the magnetic field, and the angle between the direction of the electric field's polarization and the z-axis, respectively. ω is the angular frequency of the applied AC field (i.e., light field). Here, $\mathcal{A} = 0$ for the linearly polarized light, which indicates that there is no vector component; otherwise, $\mathcal{A} = 1$ for the right-handed and $\mathcal{A} = -1$ for the left-handed circularly polarized light. In the absence of a magnetic field (or in a weak magnetic field regime) such as OPM operating in the SERF regime, we can select $\cos(\theta_k) = \cos(\theta_p) = 1$. Here, m_J independent factors $\alpha_v^0(\omega)$ is the scalar polarizability component leading the SLSs; $\alpha_v^1(\omega)$, the vector polarizability component leading to the VLSs; and $\alpha_v^2(\omega)$, the tensor polarizability component leading to the TLSs. Considering the reduced matrix elements of the dipole operator, they are expressed as follows:[13]

$$\alpha_v^0(\omega) = \frac{1}{3\,(2J_v+1)}\sum_{J_k} |\langle\psi_v\|D\|\psi_k\rangle|^2 \times \frac{\omega - \omega_{kv}}{(\omega-\omega_{kv})^2 + (2\pi\times\Gamma_{D1}/2)^2}, \quad \text{(S16)}$$

$$\alpha_v^1(\omega) = -\sqrt{\frac{6J_v}{(J_v+1)(2J_v+1)}} \sum_{J_k} \begin{Bmatrix} J_v & 1 & J_v \\ 1 & J_k & 1 \end{Bmatrix} \times (-1)^{J_v+J_k+1} \left|\langle \psi_v \| D \| \psi_k \rangle\right|^2 \times \frac{\omega - \omega_{kv}}{(\omega-\omega_{kv})^2 + (2\pi \times \Gamma_{D1}/2)^2}, \tag{S17}$$

$$\alpha_v^2(\omega) = -2\sqrt{\frac{5J_v(2J_v-1)}{6(J_v+1)(2J_v+1)(2J_v+3)}} \sum_{J_k} \begin{Bmatrix} J_v & 2 & J_v \\ 1 & J_k & 1 \end{Bmatrix} \times (-1)^{J_v+J_k+1} \left|\langle \psi_v \| D \| \psi_k \rangle\right|^2 \times \frac{\omega - \omega_{kv}}{(\omega-\omega_{kv})^2 + (2\pi \times \Gamma_{D1}/2)^2} \tag{S18}$$

where J_v and J_k are the angular momentum of the ground state and intermediate states allowed by the dipole selection rules (D1 line of Rb in our study due to a laser with λ=795 nm is utilized). $\omega_{kv} = \omega_k^0 - \omega_v^0$ and $|\langle \psi_v \| D \| \psi_k \rangle|$ is the reduced matrix element indicating electric dipole amplitude between the $|\psi_v\rangle$ and $|\psi_k\rangle$ states. Γ_{D1} is the transition's full-width half maximum (FWHM) in non-angular units.

In addition to the AC Stark shift owing to the "virtual transition" described above, there is another type of light shift known as the "real transition" shift.[6] While an atom stays in an excited state after absorbing a photon, the electron spin becomes randomized due to rapid collisional mixing. However, the hyperfine interaction is too weak for the nuclear spin to become depolarized in a short period of time before the atom decays back to its ground state. Hence, the nuclear spin remains coherent before and after the excitation. Generally, the gyromagnetic ratio differs between the ground and excited states; thus, the spin precession acquires a phase difference relative to the atoms not excited during the period. The result is a shift in the Zeeman transition frequency, but this effect is typically much smaller than the precession frequency itself, so it is not a concern in OPM operating in the SERF regime.

SLSs are common shifts of the net (or center of mass) ground-state energy or the hyperfine structure.[14] It is evident that the SLSs are identical for all the two magnetic states ($m_J = \pm 1/2$). Notably, the SERF OPM is insensitive to the overall energy shift $\delta E_v^0(\omega)$ since the ground-state hyperfine structure cannot be resolved due to the high buffer gas pressure in our vapor cells (pressure shift is about on the order of several GHz). Moreover, VLSs are linearly correlated with the magnetic momentum m_J and will be opposite for the $m_J = +1/2$ and $m_J = -1/2$ states. It mimics the behavior of a magnetic field and modifies both the precession frequency and orientation of

free spins, thereby transforming AC Stark shifts to Zeeman shifts. Note that under normal operating conditions for SERF OPM, the VLSs are typically on the order of nT and must be considered when the magnitude of the ambient magnetic field is comparable. TLSs are normally viewed as an effective electric field gradient that interacts with the electric quadrupole moment of the atom. TLSs are associated with linearly polarized light and result in quadratic shifts of the magnetic sublevels.[14] As a consequence of its quadratic dependence on the magnetic quantum number m_J, TLSs are identical for both magnetic states ($m_J = \pm 1/2$) and do not change the overall structure of sub-energy levels. In conclusion, in Rb OPM operating in the SERF regime, only the VLSs could be taken into account in the D1 transition of Rb and usually expressed as follows in terms of light frequency ν:[15]

$$L_{vector} = -\frac{I}{h\nu_p}\frac{r_e f_{D1} c}{\gamma_e}\frac{\nu - \nu_{D1}}{(\nu - \nu_{D1})^2 + (\Gamma_{D1}/2)^2}\mathcal{A} \tag{S19}$$

Where I is the light intensity, $h\nu_p$ represents the energy of a single photon, the gyromagnetic ratio of electron γ_e is given by $\gamma_e = g_J \mu_B/\hbar$, r_e is the classical electron radius. f_{D1} is the oscillator strength which could be approximately given by $1/3$ for D1 line of Rb, respectively.

Supplementary Note 6. Spectroscopic ellipsometry characterization and optical properties of amorphous silicon (a-Si) film

An amorphous silicon (a-Si) layer with a thickness of 600 nm is deposited on a silica wafer by plasma-enhanced chemical vapor deposition (PECVD). The silica substrate has a thickness of 500 μm. For the purpose of determining the optical properties of the a-Si film, a reflection-mode spectroscopic ellipsometry (J.A. Woollam M-2000) is employed.[16] The measurements are taken at an angle of incidence θ_i = 60° with respect to the normal plane of the a-Si layer. The dielectric function of the film is modeled by a Cody-Lorentz oscillator. The measured and modeled Psi (Ψ) and Delta (Δ) curves are displayed in Figure S3a, demonstrating a strong agreement between the measured and modeled data, as evidenced by a relatively low mean-squared-error for the fit (MSE = 15.339). The corresponding results for the extracted values of refractive index n and extinction coefficient k are plotted in Figure S3b. The deposited a-Si film exhibits a high refractive index n ($>$ 3.5) as well as negligible absorption coefficient k ($\sim$ 0) over the short-wavelength near-infrared (SWIR) region, which means that it is suitable for chip-scale atomic sensors with various sensitive sources, such as K, Rb, Cs. Specifically, $n = 3.614$, $k = 0$ at a wavelength of 795 nm, corresponding to the D1 transition wavelength of Rb.

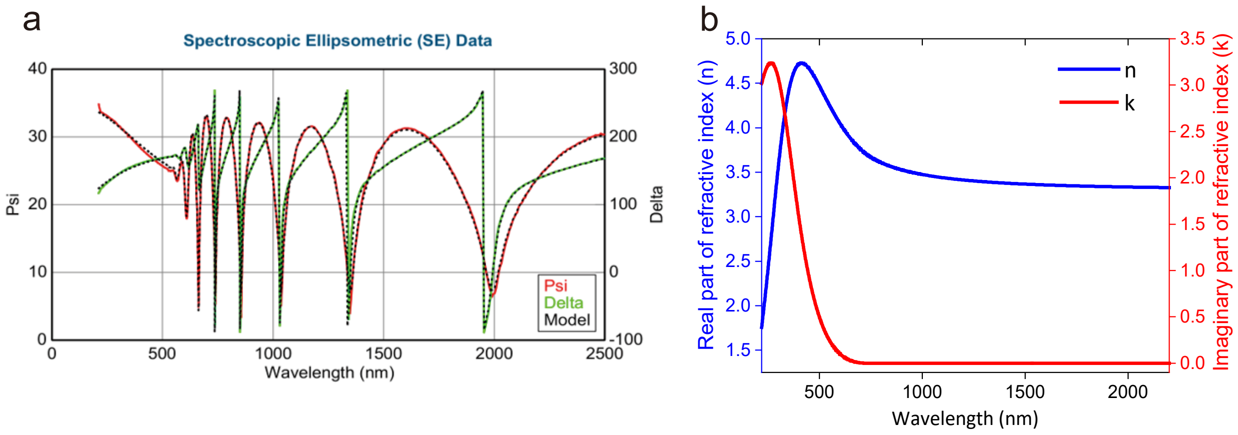

Figure S3: Optical characterization of a-Si film. (a) Measured and modeled Psi (Ψ) and Delta (Δ) curves of the film through ellipsometric characterization, displaying a close correspondence between the measured and modeled data. (b) Refractive index n and extinction coefficient k of the a-Si film, measured with the help of spectroscopic ellipsometry.

References

1 R. J. Potton, "Reciprocity in optics," *Reports on Progress in Physics* **67**(5), 717 (2004).

2 C. Menzel, C. Rockstuhl, and F. Lederer, "Advanced jones calculus for the classification of periodic metamaterials," *Physical Review A* **82**(5), 053811 (2010).

3 A. Kumar and A. Ghatak, *Polarization of light with applications in optical fibers*, SPIE (2011).

4 R. Chipman, W. S. T. Lam, and G. Young, *Polarized light and optical systems*, CRC press (2018).

5 Z. Huang, Y. Zheng, J. Li, *et al.*, "High-resolution metalens imaging polarimetry," *Nano Letters* **23**(23), 10991–10997 (2023).

6 S. Appelt, A. B.-A. Baranga, C. Erickson, *et al.*, "Theory of spin-exchange optical pumping of 3he and 129xe," *Physical Review A* **58**(2), 1412 (1998).

7 S. J. Seltzer, *Developments in alkali-metal atomic magnetometry*. PhD thesis, Princeton University (2008).

8 J. Allred, R. Lyman, T. Kornack, *et al.*, "High-sensitivity atomic magnetometer unaffected by spin-exchange relaxation," *Physical Review Letters* **89**(13), 130801 (2002).

9 H. Dang, A. C. Maloof, and M. V. Romalis, "Ultrahigh sensitivity magnetic field and magnetization measurements with an atomic magnetometer," *Applied Physics Letters* **97**(15) (2010).

10 M. Ledbetter, I. Savukov, V. Acosta, *et al.*, "Spin-exchange-relaxation-free magnetometry with cs vapor," *Physical Review A* **77**(3), 033408 (2008).

11 B. Mathur, H. Tang, and W. Happer, "Light shifts in the alkali atoms," *Physical Review* **171**(1), 11 (1968).

12 Q.-Q. Hu, C. Freier, Y. Sun, *et al.*, "Observation of vector and tensor light shifts in rb 87 using near-resonant, stimulated raman spectroscopy," *Physical Review A* **97**(1), 013424 (2018).

13 B. Arora and B. Sahoo, "State-insensitive trapping of rb atoms: Linearly versus circularly polarized light," *Physical Review A* **86**(3), 033416 (2012).

14 W. Happer and B. Mathur, "Effective operator formalism in optical pumping," *Physical Review* **163**(1), 12 (1967).

15 J. Lee, *New Constraints on the Axion's Coupling to Nucleons from a Spin Mass Interaction Limiting Experiment (SMILE)*. PhD thesis, Princeton University (2019).

16 J. N. Hilfiker, N. Singh, T. Tiwald, *et al.*, "Survey of methods to characterize thin absorbing films with spectroscopic ellipsometry," *Thin Solid Films* **516**(22), 7979–7989 (2008).
